# Dressing-Change Frequency in Acute Hand Burns: A Narrative Review of Healing, Infection Risk, Pain, Scar Quality, and Functional Recovery

**DOI:** 10.7759/cureus.112804

**Published:** 2026-07-16

**Authors:** Amta Azeem, Aviral C Sharma, Jose M Garchitorena, Ibrahim H Omari

**Affiliations:** 1 Medicine, Ponce Health Sciences University, St. Louis, USA; 2 General Practice, Sheba Medical Center, Ramat Gan, ISR

**Keywords:** biobrane, burn dressings, burn rehabilitation, dressing change, functional recovery, hand burns, partial-thickness burns, scar quality, silver dressings, wound healing

## Abstract

Acute hand burns require dressing strategies that support wound healing while preserving motion, splint tolerance, pain control, scar quality, and functional recovery. However, the optimal dressing-change frequency for acute hand burns remains poorly defined because most available studies evaluate dressing type rather than dressing interval, and few report hand-specific outcomes. This narrative review synthesizes evidence from partial-thickness burn dressing studies, silver and biosynthetic dressing literature, hand-burn rehabilitation research, scar-outcome studies, and burn pain and infection literature. Current evidence suggests that modern occlusive, antimicrobial, silicone foam, synthetic, and biosynthetic dressings may reduce dressing-change burden and procedural pain in selected superficial or partial-thickness burns while maintaining acceptable healing outcomes. However, extended dressing intervals require careful patient and wound selection, particularly when burn depth is uncertain, exudate is heavy, dressing adherence is poor, infection is suspected, or follow-up reliability is limited. For hand burns, dressing frequency should be individualized not only according to wound status but also according to functional needs, including early range of motion, splint compatibility, edema control, and therapy participation. Overall, the available literature does not support routine daily dressing changes for every acute hand burn, but stronger hand-specific comparative studies are needed to define optimal dressing-change intervals using standardized healing, infection, pain, scar, and functional outcomes.

## Introduction and background

Acute hand burns represent a clinically important subset of burn injuries because even relatively small wounds can cause substantial long-term morbidity when healing, motion, or scar maturation is impaired. The American Burn Association recommends burn center consultation or transfer consideration for deep partial-thickness or full-thickness burns involving functionally sensitive areas such as the hands, underscoring the importance of early specialist assessment in these injuries [1]. Partial-thickness burns extend through the epidermis into the dermis, superficial partial-thickness burns involve the upper dermis and generally heal more readily, and deep partial-thickness burns extend further into the dermis and carry greater risks of delayed healing and scarring. Because the hand contains closely integrated skin, tendons, joints, neurovascular structures, and intrinsic muscles, acute management must prioritize not only wound closure but also preservation of range of motion, dexterity, grip strength, and independence in activities of daily living [2].

The goals of acute hand-burn care include accurate assessment of burn depth, prevention of infection, maintenance of a moist wound environment, edema control, pain reduction, splinting when indicated, and early rehabilitation [2,3]. Early therapy is particularly important because burn injury to the hand can compromise function through pain-limited movement, edema, stiffness, hypertrophic scarring, and contracture formation [3,4]. In a clinical series of patients with hand burns, Kara et al. emphasized that physiotherapy and occupational therapy beginning at admission were important components of restoring functional independence after hand-burn trauma [4]. Hand-burn rehabilitation literature similarly highlights that functional recovery depends on coordinated wound care, positioning, splinting, scar management, and progressive mobilization throughout the acute and recovery phases [3,5].

Dressing selection is a central component of acute burn wound care, yet the optimal dressing-change interval for hand burns remains poorly defined. Traditional topical regimens, particularly those using silver sulfadiazine with gauze, often require frequent dressing changes to reapply medication and reassess the wound. However, frequent dressing changes may increase procedural pain, anxiety, resource use, and mechanical disruption of newly forming epithelium. These concerns are especially relevant for hand burns, where pain and bulky dressings may interfere with splinting, active motion, and participation in therapy. In contrast, modern occlusive, antimicrobial, and biosynthetic dressings are designed to remain in place for longer intervals while maintaining a moist wound environment and reducing dressing-related trauma [6]. Biosynthetic dressings are temporary wound coverings composed of synthetic materials, sometimes combined with biologic components, that protect the wound and support re-epithelialization.

Evidence from partial-thickness burn studies suggests that advanced dressings may reduce dressing-change burden and improve patient comfort compared with conventional silver sulfadiazine-based care. A Cochrane review of dressings for superficial and partial-thickness burns concluded that available trials are heterogeneous and often methodologically limited, but that dressing type may influence healing, pain, and care burden [6]. In a randomized trial, Caruso et al. compared a silver-containing hydrofiber dressing with silver sulfadiazine in partial-thickness burns, supporting the role of advanced dressings as alternatives to conventional silver sulfadiazine-based care [7]. Muangman et al. similarly found that silver-containing hydrofiber dressings were associated with decreased pain, improved convenience, and favorable healing outcomes compared with 1% silver sulfadiazine in partial-thickness burns [8].

Despite these findings, direct hand-specific evidence regarding dressing-change frequency remains limited because most available studies compare dressing materials rather than evaluating change frequency as an independent variable. Furthermore, many burn dressing trials are not specific to the hand and do not consistently report hand-centered outcomes such as range of motion, grip strength, return to activity, splint adherence, therapy participation, or validated scar quality measures. Consequently, it remains unclear whether daily dressing changes are necessary for acute hand burns or whether extended dressing intervals can provide comparable wound surveillance while improving comfort and functional recovery. This lack of hand-specific comparative evidence represents the principal knowledge gap addressed by this review.

This narrative review examines the available evidence surrounding dressing-change frequency in acute hand burns, with attention to healing time, infection risk, scar quality, pain, and functional outcomes. Given the limited direct comparative evidence specific to acute hand burns, this review incorporates broader partial-thickness burn dressing studies as indirect evidence when they report dressing wear time, dressing-change burden, healing, infection, pain, or outpatient feasibility. These non-hand studies are not interpreted as direct evidence for hand burns but are used to contextualize dressing-change decisions in selected superficial or partial-thickness hand burns alongside hand-specific rehabilitation and dressing literature.

## Review

Methods

Review Design

This article was designed as a narrative review rather than a systematic review or meta-analysis. A narrative review format was selected because the available literature on dressing-change frequency specifically in acute hand burns is limited, heterogeneous, and often indirect. Many relevant studies evaluate burn dressing type, pain, healing, infection, or rehabilitation outcomes without isolating dressing-change frequency as the primary exposure. Therefore, the purpose of this review was to synthesize clinically relevant evidence from hand-burn management, partial-thickness burn dressing studies, wound-healing literature, scar management, and burn rehabilitation to evaluate how dressing-change frequency may influence healing, infection risk, pain, scar quality, and functional recovery.

Literature Search Strategy

A focused narrative literature search was conducted in PubMed and Google Scholar through June 2026 to identify clinically relevant evidence related to acute hand burns, partial-thickness burn dressings, dressing wear time, pain, infection, scarring, and functional recovery. The specific search queries used for each platform are provided in Appendix A.

Search queries combined terms related to acute hand burns, partial-thickness burns, dressing-change frequency, dressing wear time, silver-containing dressings, hydrofiber dressings, silicone foam dressings, biosynthetic dressings, silver sulfadiazine-based care, wound healing, infection risk, procedural pain, scar outcomes, range of motion, splinting, and hand therapy.

Because direct evidence on dressing-change frequency specifically in acute hand burns was limited, broader partial-thickness burn dressing studies were included only when they provided clinically relevant information on dressing burden, healing, infection, pain, outpatient feasibility, or dressing wear time. These broader studies were treated as indirect supportive evidence rather than direct hand-burn evidence. Hand-burn rehabilitation and scar-management literature were also included to contextualize why dressing frequency may be particularly important for splint tolerance, early motion, edema control, scar quality, and functional recovery. Reference lists of relevant articles and clinical guidance documents were reviewed to identify additional sources.

Article Selection Rationale

Studies were eligible if they addressed acute hand burns or acute partial-thickness burn dressings and reported dressing wear time or change burden, healing, infection, pain, scarring, rehabilitation, or functional outcomes. Non-hand partial-thickness burn studies were retained as indirect evidence. Studies focused exclusively on chronic wounds, donor sites, non-burn traumatic wounds, animal models, or unrelated reconstructive topics were excluded unless they provided directly relevant principles for acute burn dressing management.

Article Selection

Titles, abstracts, and full texts were reviewed selectively to identify sources most applicable to the clinical question. Full texts were reviewed when the title or abstract suggested potential relevance to acute burn dressings, hand-burn care, dressing-change frequency, wound healing, infection, pain, scarring, or functional outcomes. Because this was a narrative review, articles were not selected according to a formal PRISMA-based systematic review process. Instead, emphasis was placed on identifying clinically relevant, peer-reviewed sources that could inform the relationship between dressing-change practices and outcomes in acute hand burns.

When multiple studies addressed similar concepts, higher-quality evidence such as randomized controlled trials, systematic reviews, clinical reviews, and larger observational studies was prioritized. Hand-specific literature was prioritized when available. When hand-specific evidence was limited, broader partial-thickness burn dressing studies were included only when their findings were relevant to dressing-change burden, pain, healing, infection, or outpatient dressing management.

Data Extraction and Synthesis

Information from selected sources was summarized narratively. When available, the review considered study design, patient population, burn depth, burn location, dressing type, dressing wear time, healing outcomes, infection outcomes, pain, scar outcomes, and functional measures.

Because the literature was heterogeneous and often indirect, findings were synthesized descriptively rather than pooled quantitatively. Findings were synthesized descriptively and organized by clinically relevant outcome domains: healing time, infection risk, pain, scar quality, and functional recovery. Particular attention was given to whether dressing-change frequency was directly evaluated or whether conclusions were inferred from studies on dressing type, dressing wear time, or dressing-related pain.

Approach to Evidence Interpretation

The evidence was interpreted conservatively. Studies on partial-thickness burns that were not specific to the hand were not treated as direct evidence for acute hand burns, even when they reported dressing wear time, dressing-change frequency, pain, healing, or infection outcomes. Instead, they were used to support broader principles related to burn dressing performance, dressing-change burden, pain, and healing. Similarly, rehabilitation and scar-management studies were used to contextualize why dressing frequency may be especially important for hand burns, but they were not interpreted as direct comparisons of dressing-change intervals unless such comparisons were explicitly reported.

The review distinguishes between direct evidence, such as studies that report dressing-change interval or dressing burden, and indirect evidence, such as studies showing that advanced dressings reduce pain or remain in place longer than conventional silver sulfadiazine-based regimens. This distinction was maintained to avoid overstating the strength of the literature.

Systematic reviews and randomized comparative trials were given greater interpretive weight than observational studies, technical reports, case reports, and expert reviews; however, no formal risk-of-bias tool or certainty-of-evidence grading system was applied.

Outcomes of Interest

The primary outcomes of interest were wound healing and infection risk. Healing was assessed using reported time to re-epithelialization, wound closure, or clinician-documented healing when available. Infection outcomes included wound infection, cellulitis, need for systemic antibiotics, or other infection-related complications as defined by individual studies.

Secondary outcomes included pain during dressing changes, scar quality, hypertrophic scarring, contracture, range of motion, adherence to splinting or therapy, and return to activity. Because hand burns have functional consequences beyond wound closure, outcomes related to rehabilitation and function were considered central to the review even when they were inconsistently reported across studies.

Limitations of the Review Method

This review does not provide a formal risk-of-bias assessment, certainty-of-evidence grading, or meta-analysis. The narrative approach allows integration of clinically relevant evidence across related areas of burn care but is limited by potential selection bias, heterogeneity of included studies, and reliance on indirect evidence when hand-specific dressing-frequency data are unavailable. As a result, conclusions are presented as clinically informed interpretations rather than definitive practice guidelines.

Results

Evidence Base and Relevance to Acute Hand Burns

Evidence was organized into three categories: general burn-care and wound-healing literature relevant to partial-thickness burn management [9-15], comparative dressing studies reporting healing, pain, dressing burden, dressing wear time, or outpatient feasibility [16-23], and hand-burn literature addressing splinting, rehabilitation, range of motion, and function. The comparative dressing studies included many non-hand partial-thickness burn populations and were therefore used as indirect evidence for dressing-change burden and safety considerations rather than as direct evidence for acute hand burns.

The most applicable dressing studies were partial-thickness burn trials and reviews evaluating conservative dressing strategies, including systematic reviews of pediatric and general partial-thickness burn care [16-18] and comparative studies on hydrofiber, silver, foam, and synthetic dressings [19-23]. General burn-care reviews emphasize that dressing selection should consider burn depth, wound progression, infection prevention, pain control, and outpatient feasibility [9-15], while dressing-specific comparative studies more directly report dressing-change burden, pain, cost, and healing outcomes [16-23].

Most comparative studies evaluated dressing products rather than dressing frequency alone. Silver and antimicrobial reviews emphasize that antimicrobial activity varies by product and does not uniformly predict faster healing [24-26]. Hydrofiber and silver dressings were examined in partial-thickness burn trials [16-19], Suprathel® (PolyMedics Innovations GmbH, Filderstadt, Germany), Mepilex® Ag (Mölnlycke Health Care, Gothenburg, Sweden), and soft silicone foam dressings were evaluated in outpatient or comparative partial-thickness burn studies [20-23], and Biobrane® (Smith & Nephew Medical Limited, Hull, UK) was evaluated in pediatric partial-thickness, intermediate-depth, and mid-dermal burn populations [27-34]. As a result, the findings should be interpreted as evidence regarding dressing systems that permit less frequent dressing changes, rather than as definitive evidence that frequency alone determines outcomes.

Hand-specific literature provides a strong rationale for minimizing dressing-related pain and preserving motion, although direct interval-comparison studies remain limited. Large clinical experience with acute hand burns highlights the importance of early structured management to preserve hand function [35], while reviews of hand and upper limb burns emphasize that treatment must account for burn depth, anatomic location, and motion preservation [36]. Rehabilitation-focused literature identifies edema control, positioning, splinting, range-of-motion therapy, strengthening, and scar management as core components of burned-hand recovery [37,38]. Hand-specific glove-dressing reports suggest that conformable dressings may reduce bulk around the digits and web spaces while preserving motion [39-41]. Broader burn-rehabilitation consensus literature reinforces that functional recovery requires interdisciplinary therapy, standardized outcome measurement, contracture prevention, and return to daily activity [42,43]. Therefore, the clinical importance of dressing-change frequency in hand burns lies not only in wound closure but also in whether the dressing strategy supports therapy participation and reduces dressing-related barriers to movement.

The evidence domains incorporated into this narrative synthesis, along with their key contributions and limitations, are summarized in Table 1.

**Table 1:** Evidence domains included in the narrative synthesis

Evidence domain	Main source types	Key contribution to this review	Limitations
General burn wound management	Reviews, guidelines, and consensus statements [9-15]	Establishes principles of burn-depth assessment, wound surveillance, infection prevention, outpatient triage, and conservative management of partial-thickness burns	Not specific to hand burns or dressing-change frequency
Partial-thickness burn dressing trials and reviews	Systematic reviews and comparative dressing studies [16-23]	Provides indirect evidence on healing time, dressing burden, pain, outpatient feasibility, dressing tolerance, and cost considerations for advanced dressings used in partial-thickness burns	Mostly not hand-specific; used as indirect evidence only. Dressing material, wear time, and change interval are often confounded.
Hydrofiber and silicone foam dressing evidence	Silver-containing hydrofiber and soft silicone foam studies [7,8,19,23]	Supports reduced dressing-change burden, improved comfort, acceptable healing, and reduced resource use in selected partial-thickness burns	Findings come from broader partial-thickness burn populations rather than isolated acute hand burns
Silver and antimicrobial dressing literature	Silver-focused reviews and silver-containing dressing studies [7,8,19,23-26]	Clarifies antimicrobial rationale, product variability, and limitations of assuming that silver activity consistently improves healing or infection outcomes	Infection and healing outcomes vary by product, burn depth, comparator, and study design
Biosynthetic dressing literature	Biobrane studies in pediatric, partial-thickness, intermediate-depth, and mid-dermal burns [27-34]	Provides evidence on extended wear, adherence, healing, hospitalization, pain, infection, grafting, and treatment burden for selected burns	Outcomes vary by burn depth, exudate, timing of application, comparator dressing, and patient selection
Hand-burn rehabilitation literature	Hand-burn series, hand-burn reviews, rehabilitation studies, and consensus literature [35-38,42,43]	Demonstrates why early edema control, splinting, positioning, range of motion, scar management, and therapy participation are central to functional recovery	Does not directly compare dressing-change intervals
Hand-specific glove-dressing literature	Technical reports, case reports, and early clinical reports of conformable hand dressings [39-41]	Supports the concept that glove-style dressings may reduce bulk around digits and web spaces while preserving motion or allowing easier hand use	Low-level evidence; does not establish an optimal dressing-change interval
Scar and long-term outcome literature	Hypertrophic scar risk studies and scar-scale validation studies [44-50]	Supports the relationship between delayed epithelialization and hypertrophic scarring and identifies validated scar assessment tools	Mostly indirect to dressing-change frequency; few dressing trials report long-term scar outcomes
Pain and infection context	Burn pain guidelines/reviews and burn infection reviews [51-55]	Supports attention to procedural pain, dressing-change pain, infection risk, and need for surveillance	Broad burn literature; not specific to hand burns or dressing interval trials

Dressing-Change Frequency, Dressing Wear Time, and Healing Outcomes

In partial-thickness burn literature, systematic reviews support the use of modern dressings as alternatives to silver sulfadiazine, although study quality is variable [16-18]. More directly, silver-containing hydrofiber dressings were associated with favorable healing, fewer dressing changes, or improved comfort compared with silver sulfadiazine-based care in randomized partial-thickness burn studies [7,8], while Verbelen et al. compared Aquacel® Ag (Convatec, London, UK) with Acticoat in a prospective randomized partial-thickness burn trial [19]. Suprathel and Mepilex Ag studies support outpatient partial-thickness burn care with multi-day follow-up or reduced dressing disturbance [20-22]. These findings are consistent with systematic reviews concluding that silver sulfadiazine is not clearly superior to modern dressings for superficial or partial-thickness burns and may be associated with disadvantages related to healing time, dressing burden, or wound-care experience [16-18]. Silver-specific reviews further caution that antimicrobial activity should be balanced against potential effects on healing and product-specific variability [24-26].

Hydrofiber silver dressings are particularly relevant because randomized partial-thickness burn studies comparing them with silver sulfadiazine-based care reported fewer dressing changes, improved comfort, and favorable healing outcomes [7,8]. However, silver-focused reviews emphasize that antimicrobial benefit and healing effects vary by product, and these trials were not specific to acute hand burns or designed to assess hand-centered outcomes such as active range of motion, grip strength, splint tolerance, or therapy participation [24-26].

Suprathel and Mepilex Ag studies support outpatient partial-thickness burn care with reduced wound-bed disturbance and multi-day follow-up, but these studies should be interpreted separately from Biobrane pediatric burn studies because the dressing materials, populations, and infection risks differ [20-22,27-32]. Suprathel, for example, is designed to remain adherent to the wound surface while progressively detaching as re-epithelialization occurs [20]. This approach may reduce wound-bed disruption and procedural pain, although direct product cost may be higher than some alternatives [20]. Mepilex Ag has been evaluated in outpatient partial-thickness burn protocols with multi-day follow-up [20-22], and Silverstein et al. specifically compared a silver-containing soft silicone foam dressing with silver sulfadiazine cream in partial-thickness thermal burns [23].

Biobrane has been evaluated in pediatric superficial partial-thickness, intermediate-depth, and mid-dermal burns [27-32,34], as well as in a randomized partial-thickness burn comparison with silver sulfadiazine [33]. Across these studies, reported outcomes include healing time, hospitalization, infection, grafting, dressing adherence, and treatment burden [27-34]. Some Biobrane studies reported shorter healing time or hospitalization compared with topical antimicrobial or conventional dressing regimens [28-31], whereas the mid-dermal pediatric pilot trial by Hyland et al. reported a nonsignificant trend toward faster healing but a higher numerical infection rate compared with Acticoat [27]. Depth-specific interpretation is important because the pediatric mid-dermal Biobrane trial by Hyland et al. raised concern for a higher numerical infection rate compared with Acticoat [27], while other pediatric and partial-thickness Biobrane studies reported more favorable healing or resource-use outcomes in selected wounds [28-34]. These findings suggest that extended-wear biosynthetic dressings should be used selectively, with closer reassessment when burn depth, exudate burden, adherence, or infection risk is uncertain [27-34].

Overall, the evidence most strongly supports less frequent dressing changes in selected superficial or partial-thickness burns when modern dressings have demonstrated acceptable healing, pain, and outpatient feasibility in comparative studies [16-22]. For biosynthetic dressings, patient selection is particularly important because Biobrane outcomes differ between superficial pediatric scald studies and deeper mid-dermal burn studies [27-32]. For deeper partial-thickness hand burns, wounds with heavy exudate, uncertain depth, poor dressing adherence, or concern for infection, more frequent reassessment may be necessary despite the use of longer-wear dressings; this is supported by general burn-care principles [9-15], silver-dressing limitations [24-26], variable Biobrane infection findings [27-34], and burn infection surveillance literature [54,55].

The dressing categories and representative studies most relevant to reducing dressing-change burden are summarized in Table 2.

**Table 2:** Dressing studies most relevant to reduced dressing-change burden

Dressing category	Representative studies	Dressing-change relevance	Healing and safety findings	Relevance to acute hand burns
Hydrofiber silver dressings	Caruso et al.; Muangman et al.; Verbelen et al. [7,8,19]	Designed for multi-day wear; compared with silver sulfadiazine in some trials and with other silver dressings in others	Caruso et al. and Muangman et al. reported reduced dressing-change burden, improved comfort or pain outcomes, and favorable healing compared with silver sulfadiazine-based care [7,8]. Verbelen et al. compared Aquacel Ag with Acticoat in partial-thickness burns [19].	Indirectly relevant for selected superficial and partial-thickness hand burns because fewer dressing changes may reduce procedural pain, but these trials were not hand-specific and should not be interpreted as direct hand-burn evidence.
Silver sulfadiazine regimens	Cochrane and systematic reviews; comparator arms in hydrofiber and foam trials [6-8,16-18,23,24]	Often requires daily or frequent reapplication and wound reassessment	Silver sulfadiazine remains a traditional comparator, but systematic reviews and comparative trials do not show consistent superiority over modern dressings and often highlight disadvantages related to dressing burden, pain, or healing experience [6-8,16-18,23,24].	May be useful when frequent inspection is needed, but daily or frequent changes may worsen procedural pain and interfere with therapy participation
Nanocrystalline silver dressings	Acticoat-related comparative studies and silver reviews [19,21,24-27]	Typically used in multi-day antimicrobial dressing protocols depending on institutional practice and wound status	Acticoat and other silver-containing dressings provide antimicrobial coverage, but healing and infection outcomes vary by product, burn depth, comparator dressing, and study design [19,21,24-27].	May be useful when antimicrobial coverage and reduced dressing frequency are desired, but hand-specific functional outcomes are rarely reported
Silicone foam dressings	Mepilex Ag/Suprathel comparative studies; soft silicone foam versus silver sulfadiazine studies [20-23]	Usually changed at multi-day intervals depending on protocol, exudate, and wound reassessment needs	Mepilex Ag has shown acceptable healing in outpatient partial-thickness burn studies [20-22], and Silverstein et al. specifically evaluated a silver-containing soft silicone foam dressing versus silver sulfadiazine cream with outcomes including cost-effectiveness, performance, tolerance, and safety [23].	Potentially useful for outpatient hand burns if the dressing conforms well and does not restrict motion, splinting, or therapy
Synthetic polymer dressings	Suprathel studies [20,22]	Applied as an adherent synthetic dressing; may remain in place with selective trimming as healing progresses	Hundeshagen et al. reported similar re-epithelialization time between Suprathel and Mepilex Ag, lower early pain with Suprathel, and favorable patient-rated appearance, although direct product cost was higher [20]. Karlsson et al. compared Suprathel and Mepilex Ag in pediatric partial-thickness burns [22].	Attractive for pain reduction and reduced wound-bed disturbance, but requires careful selection and surveillance, especially if infection risk or exudate burden is uncertain
Biosynthetic Biobrane dressings	Hyland et al.; Lal et al.; Barret et al.; Cassidy et al.; Lesher et al.; Mandal; Gerding et al.; Fan et al. [27-34]	Often intended to remain adherent until healing if applied to an appropriate wound and if adherence is maintained	Biobrane studies reported variable outcomes. Some studies found favorable healing, hospitalization, pain, cost, or treatment-burden outcomes in selected superficial or partial-thickness burns [28-34], while Hyland et al. reported a higher numerical infection rate with Biobrane than Acticoat in pediatric mid-dermal burns [27].	May be useful in selected partial-thickness burns, but infection risk, adherence, depth, and exudate must be monitored closely; evidence is not specific enough to define a universal hand-burn dressing interval
Hand-specific glove-style dressings	Tan et al.; Kok et al.; Ratliff et al. [39-41]	Designed to conform to the hand, fingers, and web spaces while reducing bulky layered dressings	Technical and case-based reports suggest that glove-style or conformable hand dressings can provide coverage while preserving motion or reducing dressing bulk [39-41].	Highly relevant conceptually for hand burns because reduced bulk may improve range of motion and splint compatibility, but evidence remains low-level and does not establish an optimal dressing-change frequency

Pain, Procedural Burden, and Patient Experience

Pain during dressing changes emerged as one of the most consistent reasons to consider less frequent dressing changes. Burn pain is multifactorial and includes background pain, breakthrough pain, procedural pain, and therapy-associated pain [51-53]. Dressing removal and reapplication are among the most painful recurring procedures in burn care, particularly in partial-thickness burns, where exposed nerve endings and repeated manipulation of the wound surface increase sensitivity [51-53]. Therefore, dressing systems that reduce dressing-change frequency or avoid disrupting newly forming epithelium may improve patient comfort, a concept supported by silver-containing hydrofiber dressing studies [7,8,19], Suprathel/Mepilex Ag outpatient data [20-22], and burn pain literature describing procedural pain as a major component of burn care [51-53].

Silver-containing hydrofiber dressing studies reported lower dressing-related pain, fewer dressing changes, or improved convenience compared with silver sulfadiazine-based care in partial-thickness burns [7,8]. This likely reflects reduced dressing removal and a moisture-retentive wound environment reported in hydrofiber dressing studies [7,8,19], while silver reviews support the antimicrobial rationale but also emphasize product-specific variability [24-26]. Reduced procedural pain may be especially important for hand burns because rehabilitation literature emphasizes early motion, splinting, and therapy participation [35-38,42,43], while burn pain guidelines describe dressing changes and therapy as major procedural pain triggers [51-53].

Suprathel may reduce wound-bed disturbance because it remains adherent to the wound surface during re-epithelialization and detaches progressively as healing occurs [20]. In this model, unhealed regions can remain relatively undisturbed while outer dressings are removed or adjusted for inspection [20]. This principle is particularly relevant to hand burns because early motion and therapy participation are central to functional recovery [35-38,42,43], and burn pain literature identifies repeated procedures as a major source of anxiety and pain burden [51-53].

Patient experience includes more than wound closure; hydrofiber silver and outpatient synthetic dressing studies report dressing-change burden, cost, or clinic-visit implications [19-22], while burn pain and infection literature highlight the need to balance patient comfort with safe wound surveillance [51-55]. Silver-containing hydrofiber dressing studies reported fewer dressing changes compared with silver sulfadiazine-based care [7,8], and outpatient Suprathel/Mepilex Ag studies reported reduced dressing disturbance or limited dressing-change requirements during follow-up [20-22]. However, product cost and total treatment cost vary by dressing type; Hundeshagen et al. reported higher direct product cost for Suprathel than Mepilex Ag [20], while Silverstein et al. evaluated cost-effectiveness for a silver-containing soft silicone foam dressing compared with silver sulfadiazine cream [23].

In acute hand burns, the functional consequences of pain are particularly significant. Hand-burn rehabilitation studies emphasize early motion, splint tolerance, and functional use [35-38,42,43], while burn pain guidelines support minimizing repeated painful procedures when clinically safe [51-53]. Therefore, the ideal hand-burn dressing strategy should minimize procedural pain [51-53], avoid excessive bulk when possible [39-41], maintain secure wound coverage, and remain compatible with supervised early motion and splinting [35-38,42,43].

Infection Risk and Wound Surveillance

The infection evidence did not support a simple conclusion that less frequent dressing changes are universally safer or more dangerous. Instead, infection risk should be interpreted by dressing category and wound context: silver reviews describe antimicrobial benefits and limitations of silver-containing products [24-26], Biobrane studies show differing infection findings by burn depth and patient selection [27-32], and burn infection reviews emphasize host factors, wound depth, microbial burden, and surveillance [54,55]. Burn wounds are susceptible to colonization and infection because of loss of the epithelial barrier, devitalized tissue, altered local immunity, and exposure to hospital or environmental organisms [54-55]. Repeated dressing changes may increase procedural manipulation and patient discomfort, which is why modern dressing studies often report dressing-change burden, wear time, and pain alongside healing outcomes [7,8,19-23].

Silver-containing dressings are intended to reduce bacterial burden, but reviews of silver in wound care emphasize that silver activity and clinical benefit vary across products and indications [24-26]. In partial-thickness burn trials, silver dressings showed variable healing and infection outcomes depending on comparator, depth, and dressing protocol [19-22]. Silver reviews support selective use when bacterial burden or infection risk is clinically relevant, while also cautioning that silver products differ in release profile, cytotoxicity concerns, and clinical performance [24-26]. This is important in hand burns because rehabilitation literature emphasizes the need to preserve motion and splint compatibility [35-38,42,43], while silver reviews caution against assuming uniform benefit across all silver-containing products [24-26].

Biobrane studies demonstrated variable infection findings depending on burn depth and comparator dressing [27-32]. Lal et al. reported faster healing and no difference in systemic antibiotic use or infectious readmissions with Biobrane in selected superficial pediatric scald burns [28], while Barret et al. and Lesher et al. also support Biobrane as an option in selected pediatric partial-thickness burns [29,31]. However, in the pediatric mid-dermal burn pilot trial by Hyland et al., Biobrane was associated with a higher numerical infection rate than Acticoat, although the study was small and underpowered [27]. These conflicting findings suggest that Biobrane may be more appropriate in selected superficial or intermediate-depth pediatric burns than in deeper mid-dermal wounds with higher exudate or infection risk [27-32].

For acute hand burns, this means dressing-change intervals should be individualized. Less frequent dressing changes may be reasonable for clean superficial or selected partial-thickness wounds when comparative dressing studies support multi-day wear and acceptable healing [19-22] and when biosynthetic dressings are used in appropriately selected wounds with good adherence and close follow-up [27-32]. More frequent inspection may be appropriate when burn depth is uncertain, the wound is circumferential, exudate is heavy, dressing adherence is poor, pain is worsening, erythema is spreading, systemic symptoms occur, or the patient has risk factors for infection; this approach is consistent with general burn-care principles [9-15], silver and biosynthetic dressing limitations [24-32], and burn infection surveillance literature [54,55].

Scar Quality, Healing Time, and Functional Recovery

Scar outcomes were underreported in dressing-frequency studies. However, broader burn-scar literature links delayed epithelialization with hypertrophic scar risk [44,47], identifies burn depth and other clinical variables as risk factors for pathologic scarring [45,46], and supports validated scar scales for standardized outcome assessment [48-50]. These findings are relevant because dressing studies report differences in healing time, pain, and wound-bed disturbance [19-22], while scar studies show that delayed healing is associated with worse scar outcomes [44,47]. However, no hand-burn trial has directly shown that reducing dressing-change frequency independently improves long-term scar quality.

Validated scar instruments, including the Vancouver Scar Scale and Patient and Observer Scar Assessment Scale, are widely used to assess vascularity, pigmentation, pliability, thickness, relief, symptoms, and patient perception [48-50]. Among the dressing studies reviewed, scar and patient-rated appearance outcomes were most clearly reported in the Suprathel versus Mepilex Ag trial [20], whereas the broader scar literature supports Vancouver Scar Scale and Patient and Observer Scar Assessment Scale use for standardized scar assessment [48-50]. In the prospective Suprathel versus Mepilex Ag trial, patient-rated overall appearance favored Suprathel, whereas observer-rated scar parameters were less consistently different [20]. Therefore, scar quality should be included as an outcome in future hand-burn dressing studies, but current evidence does not prove that less frequent dressing changes independently reduce hypertrophic scarring in acute hand burns [20,44-50].

Functional recovery is related to wound closure but should be assessed as a separate outcome. Sheridan et al. demonstrated through a large 10-year experience with acute hand burns that structured early management is important for preserving hand function [35], while Smith et al. emphasized that treatment decisions for hand and upper-limb burns must account for burn depth, anatomic location, and motion preservation [36]. Rehabilitation-focused literature further shows that patients may experience stiffness, contracture, reduced grip strength, impaired dexterity, and difficulty with activities of daily living even after epithelialization, supporting the need for early therapy, splinting, edema control, positioning, scar management, and progressive mobilization [37,38]. Broader burn-rehabilitation consensus literature also reinforces the importance of interdisciplinary rehabilitation, standardized functional assessment, contracture prevention, and return to daily activity [42,43]. Thus, dressing protocols should be evaluated by whether they permit motion and therapy participation, not only by whether they close the wound.

Hand-specific dressing evidence remains limited. Glove-style and conformable hand dressings have been described as methods to reduce bulk around the fingers and web spaces, with technical and case-based reports suggesting improved ease of motion or preservation of range of motion [39-41]. However, the evidence for glove-style hand dressings consists mostly of technical reports, case reports, or early clinical experience rather than high-quality comparative trials [39-41]. The available hand-burn rehabilitation literature supports the principle that dressings should be compatible with early therapy [35-38,42,43], while hand-specific dressing reports support the feasibility of less bulky glove-style approaches but do not establish an optimal dressing-change interval [39-41].

Discussion

The central implication of this review is not that one dressing-change schedule should replace another, but that dressing frequency in acute hand burns should be treated as a component of the overall wound-management strategy. Existing studies rarely separate the effect of change frequency from the properties of the dressing itself, including moisture retention, antimicrobial activity, adherence, exudate control, and recommended wear time. Consequently, the available evidence supports an individualized approach rather than a universal recommendation for daily or extended-interval dressing changes. This distinction is particularly important because broader burn-dressing evidence cannot be applied directly to the hand without considering its unique functional demands.

Modern dressings may allow the wound to remain undisturbed for longer periods, but their clinical value extends beyond simply reducing the number of dressing changes. Randomized studies on silver-containing hydrofiber dressings reported fewer dressing changes, reduced pain, or improved convenience compared with silver sulfadiazine-based care, although these benefits cannot be attributed to dressing-change frequency alone [7,8]. Aquacel Ag and Acticoat have also been compared directly, demonstrating that outcomes may differ among silver-containing dressing systems [19]. One randomized outpatient trial found similar time to re-epithelialization with Suprathel and Mepilex Ag, while Suprathel was associated with lower early pain and higher direct product cost [20]. A pediatric case-control study found no significant differences in healing time, infection, operative intervention, or number of dressing changes between the two dressings [22]. A separate ambulatory trial compared Biobrane, Acticoat, Mepilex Ag, and Aquacel Ag with emphasis on re-epithelialization and cost-effectiveness [21]. Cost-effectiveness and treatment tolerance should also be considered because a silver-containing soft silicone foam dressing was evaluated against silver sulfadiazine for effectiveness, safety, tolerance, and cost-effectiveness [23].

These findings must be interpreted cautiously because most dressing studies involve partial-thickness burns at multiple anatomical sites rather than isolated hand burns. The hand differs from other body regions because successful treatment depends not only on wound closure but also on preservation of tendon glide, joint mobility, web-space depth, dexterity, and grip strength. Acute hand-burn management therefore requires continuous coordination between wound care and rehabilitation rather than sequential treatment in which therapy begins only after epithelialization [2,5]. Large clinical experience and review literature on acute hand burns support structured early management aimed at preserving hand function [35,36].

Dressing frequency may influence recovery when dressing removal, reapplication, or excessive bulk interferes with early rehabilitation. Hand-burn rehabilitation literature emphasizes positioning, edema control, splinting, range-of-motion exercises, and progressive functional use as components of recovery [37,38]. Broader rehabilitation guidance similarly emphasizes that recovery should be evaluated through function, independence, and participation rather than wound closure alone [42,43]. Accordingly, a dressing that heals the wound but restricts motion, disrupts splinting, or discourages therapy may be clinically less suitable for the hand than a comparably effective dressing that supports early movement.

Pain is an important link between dressing practices and functional recovery. Dressing changes are a recurring source of procedural pain, while movement and therapy may add further pain during the acute phase [51-53]. Reducing unnecessary dressing manipulation may improve comfort, but the resulting benefit should not be attributed solely to the interval between changes. Pain may also be influenced by dressing adherence, wound hydration, burn depth, analgesic use, and the degree of tissue disruption during removal. This confounding is evident in hydrofiber and synthetic-dressing studies, in which dressing material and change frequency were modified simultaneously [7,8,20]. Future studies should therefore distinguish pain associated with the wound itself from pain occurring specifically during dressing removal, reapplication, and rehabilitation.

In hand burns, pain has implications beyond patient comfort. Guarding and fear of movement may reduce active participation in therapy, interfere with splint tolerance, and contribute to progressive stiffness. A longer-wear dressing may therefore have functional value when it reduces repeated painful procedures without compromising wound assessment. However, extended intervals should not be pursued solely to minimize pain when clinical findings indicate the need for earlier inspection. The preferred strategy should balance procedural comfort with adequate monitoring and uninterrupted rehabilitation.

Infection surveillance remains the most important consideration limiting prolonged dressing wear. Silver-containing products should not be treated as interchangeable because reviews report variable and sometimes conflicting effects on antimicrobial activity, wound healing, and cellular toxicity [24-26]. The presence of silver also does not eliminate the need for reassessment when pain increases, exudate changes, adherence fails, or surrounding erythema develops. Therefore, an antimicrobial dressing should be viewed as one part of infection prevention rather than as justification for an automatically extended dressing interval.

The Biobrane literature further illustrates why dressing interval cannot be separated from wound selection. Selected pediatric studies reported faster healing or shorter hospitalization with Biobrane than with conventional treatment [28,29]. Lal et al. additionally reported no increased infection risk in their study population [28]. However, less favorable infection findings in deeper mid-dermal burns indicate that conclusions from superficial wounds should not be generalized to burns with greater depth, exudate, or tissue injury [27]. Additional Biobrane studies evaluated healing, cost, and clinical effectiveness in pediatric intermediate-thickness or partial-thickness burns [30-32]. Collectively, these findings support cautious, wound-specific selection rather than a universal extended-wear approach. Thus, patient and wound selection may be more important than the intended wear duration itself.

General burn-care principles support this individualized interpretation. Burn wounds may evolve after the initial assessment, particularly when depth is uncertain or local tissue injury progresses [9-11]. Practice guidelines also emphasize that wound management should reflect burn depth, location, infection risk, healing potential, and available follow-up rather than follow a single fixed protocol [14,15]. In practical terms, more frequent inspection may be warranted when the wound is heavily exudative, the dressing is lifting, burn depth is unclear, pain is worsening, or infection is suspected. Longer intervals may be appropriate when the wound is clean and superficial, the dressing remains intact, exudate is controlled, and the patient can return promptly if clinical changes occur.

The hand-specific glove-dressing literature introduces another consideration that is often absent from general burn trials: dressing configuration. Conformable glove or finger-sleeve approaches may reduce bulk around the digits and web spaces while maintaining coverage [39-41]. Although these reports do not establish an optimal change interval, they suggest that dressing design may affect motion independently of wear time. Future comparisons should therefore evaluate not only how often a dressing is changed but also whether its shape, thickness, and fixation allow tendon glide, web-space positioning, splint application, and active hand use.

Scar outcomes remain difficult to connect directly to dressing-change frequency. Delayed epithelialization is associated with a greater risk of hypertrophic scar development [44,47]. Scar formation is multifactorial and is also influenced by patient- and injury-related characteristics [45,46]. Additional studies have identified multiple clinical and demographic contributors to pathologic scarring, making it unlikely that dressing frequency alone determines long-term scar quality [45,46]. Nevertheless, avoiding unnecessary wound disruption may be clinically relevant when it supports timely epithelialization and reduces inflammation, although this relationship has not been established specifically in acute hand burns.

Scar assessment is especially important in hand-burn research because scar thickness, pliability, symptoms, and contracture may affect motion even after complete wound closure. The Vancouver Scar Scale and Patient and Observer Scar Assessment Scale offer structured methods for assessing these outcomes [48,49]. However, burn-scar instruments vary in measurement quality and may not capture all functionally important features of the hand [50]. Future studies should therefore combine validated scar measures with range of motion, grip strength, dexterity, splint adherence, and patient-reported hand function.

A clinically useful framework should consider wound depth, exudate, dressing integrity, infection risk, pain, follow-up reliability, and rehabilitation requirements together. Daily changes may be appropriate when frequent reassessment is necessary, but routine daily manipulation may impose avoidable pain and treatment burden in a stable superficial or partial-thickness wound. Conversely, a modern dressing designed for extended wear should not remain in place solely because the manufacturer permits a longer interval. The actual interval should be shortened when the wound or patient’s condition requires closer evaluation.

The evidence base has several important limitations. Dressing material and change frequency are commonly confounded, preventing determination of whether observed differences arise from the interval itself or from antimicrobial activity, moisture balance, adherence, cushioning, or exudate management. Studies also vary in burn depth, anatomical location, age group, outpatient versus inpatient treatment, comparator regimens, and outcome definitions. Most importantly, hand-centered outcomes are rarely reported, limiting the clinical translation of general partial-thickness burn studies. The narrative design and absence of formal risk-of-bias assessment further mean that the conclusions should be interpreted as clinically informed rather than definitive.

Future trials should compare predefined dressing-change intervals within the same dressing category so that the effect of frequency can be evaluated independently of material. Studies should stratify patients by burn depth and should report wound healing, infection, procedural pain, analgesic requirements, unplanned dressing changes, clinic visits, and cost. For hand burns, these outcomes should be accompanied by active range of motion, grip strength, edema, splint tolerance, therapy participation, scar quality, dexterity, and patient-reported function. Reporting whether rehabilitation was performed while the dressing remained in place would also help determine whether longer-wear dressings meaningfully support early movement.

Overall, current evidence does not justify routine daily dressing changes for every acute hand burn, nor does it support prolonged wear for every wound. Modern dressings may reduce procedural burden and permit less frequent changes in appropriately selected superficial or partial-thickness burns, but their use must remain responsive to wound evolution, infection risk, adherence, exudate, follow-up, and rehabilitation needs. The most appropriate approach is an individualized dressing interval that preserves adequate surveillance while minimizing unnecessary pain, disruption, and interference with hand function. Direct hand-specific comparative trials are required before a standardized dressing-change schedule can be recommended.

## Conclusions

The available evidence supports an individualized rather than fixed approach to dressing-change frequency in acute hand burns. For appropriately selected superficial or partial-thickness injuries, modern extended-wear dressings may reduce procedural pain, treatment burden, and unnecessary wound disruption while supporting outpatient care and early rehabilitation. These potential benefits must be balanced against the need for timely reassessment when burn depth is uncertain, exudate is heavy, dressing adherence is poor, or infection is suspected. Because most existing studies involve non-hand-burn populations and evaluate dressing systems rather than change frequency alone, definitive hand-specific recommendations cannot yet be made. Until direct comparative trials are available, the optimal strategy is the one that preserves wound surveillance while prioritizing healing, pain control, early motion, splint compatibility, scar prevention, and long-term hand function.

## Appendices

Appendix A

Search queries used in PubMed and Google Scholar

A focused literature search was conducted in PubMed and Google Scholar through June 2026. Searches were organized by topic because the literature relevant to dressing-change frequency in acute hand burns includes hand-specific management studies, broader partial-thickness burn dressing studies, rehabilitation literature, pain and infection studies, and scar-outcome research. Search terms were adapted to the syntax of each database. Reference lists of relevant reviews, guidelines, and included articles were also screened for additional sources.

PubMed Search Queries

1. Hand Burns and Dressing Practices

("hand burn" OR "hand burns" OR "burned hand" OR "upper extremity burn")

AND

(dressing OR dressings OR "dressing change" OR "dressing frequency" OR "dressing interval" OR "wear time")

2. Partial-Thickness Burn Dressings

("partial-thickness burn" OR "partial thickness burn" OR "second-degree burn" OR "superficial burn")

AND

(dressing OR dressings OR "wound coverage" OR "wound care")

AND

(healing OR infection OR pain OR "dressing change")

3. Silver and Antimicrobial Dressings

(burn OR burns)

AND

("silver dressing" OR "silver-containing dressing" OR hydrofiber OR "Aquacel Ag" OR Acticoat OR "silver sulfadiazine")

AND

(healing OR infection OR pain OR cost OR "dressing changes")

4. Advanced and Biosynthetic Dressings

(burn OR burns)

AND

(Suprathel OR "Mepilex Ag" OR Biobrane OR "silicone foam" OR "biosynthetic dressing" OR "synthetic dressing")

AND

(healing OR infection OR pain OR outpatient OR "wear time")

5. Hand Burn Rehabilitation and Function

("hand burn" OR "hand burns" OR "burned hand")

AND

(rehabilitation OR physiotherapy OR "occupational therapy" OR splinting OR "range of motion" OR "hand function" OR contracture OR scar)

Google Scholar Search Queries

The following phrases were entered separately into the Google Scholar search bar:

1. "acute hand burns" "dressing change frequency"

2. "partial thickness burns" "dressing wear time"

3. "Aquacel Ag" "silver sulfadiazine" burns

4. Suprathel "Mepilex Ag" burns

5. Biobrane burns healing infection

6. "hand burn rehabilitation" splinting

7. "glove dressing" hand burns

8. "burn dressing changes" pain

9. "burn wound infection" dressings

10. "healing time" hypertrophic scarring burns

11. "Vancouver Scar Scale" burns

12. "Patient and Observer Scar Assessment Scale" burns

Supplementary Search Procedures

Reference lists of relevant systematic reviews, randomized trials, clinical reviews, practice guidelines, and hand-burn rehabilitation articles were manually screened to identify additional eligible studies. When an article was identified through a reference list, its title was searched directly in PubMed or Google Scholar to locate the full citation and assess its relevance. No formal PRISMA-based systematic search or meta-analysis was performed.

## References

[1] (2026). Guidelines for Burn Patient Referral. American Burn Association.

[2] Soni A, Pham TN, Ko JH (2017). Acute management of hand burns. Hand Clin.

[3] Sorkin M, Cholok D, Levi B (2017). Scar management of the burned hand. Hand Clin.

[4] Kara S, Seyhan N, Öksüz S (2023). Effectiveness of early rehabilitation in hand burns. Ulus Travma Acil Cerrahi Derg.

[5] Cowan AC, Stegink-Jansen CW (2013). Rehabilitation of hand burn injuries: current updates. Injury.

[6] Wasiak J, Cleland H, Campbell F, Spinks A (2013). Dressings for superficial and partial thickness burns. Cochrane Database Syst Rev.

[7] Caruso DM, Foster KN, Blome-Eberwein SA (2006). Randomized clinical study of Hydrofiber dressing with silver or silver sulfadiazine in the management of partial-thickness burns. J Burn Care Res.

[8] Muangman P, Pundee C, Opasanon S, Muangman S (2010). A prospective, randomized trial of silver containing hydrofiber dressing versus 1% silver sulfadiazine for the treatment of partial thickness burns. Int Wound J.

[9] Greenhalgh DG (2019). Management of burns. N Engl J Med.

[10] Jeschke MG, van Baar ME, Choudhry MA, Chung KK, Gibran NS, Logsetty S (2020). Burn injury. Nat Rev Dis Primers.

[11] Rowan MP, Cancio LC, Elster EA (2015). Burn wound healing and treatment: review and advancements. Crit Care.

[12] Hettiaratchy S, Dziewulski P (2004). ABC of burns: pathophysiology and types of burns. BMJ.

[13] Singer AJ, Taira BR, Lee CC, Soroff HS (2010). Thermal burns. J Emerg Med.

[14] (2016). ISBI Practice Guidelines for Burn Care. Burns.

[15] Ji S, Xiao S, Xia Z (2024). Consensus on the treatment of second-degree burn wounds (2024 edition). Burns Trauma.

[16] Vloemans AF, Hermans MH, van der Wal MB, Liebregts J, Middelkoop E (2014). Optimal treatment of partial thickness burns in children: a systematic review. Burns.

[17] Lőrincz A, Váradi A, Hegyi P (2022). Paediatric partial-thickness burn therapy: a meta-analysis and systematic review of randomised controlled trials. Life (Basel).

[18] Heyneman A, Hoeksema H, Vandekerckhove D, Pirayesh A, Monstrey S (2016). The role of silver sulphadiazine in the conservative treatment of partial thickness burn wounds: a systematic review. Burns.

[19] Verbelen J, Hoeksema H, Heyneman A, Pirayesh A, Monstrey S (2014). Aquacel(®) Ag dressing versus Acticoat™ dressing in partial thickness burns: a prospective, randomized, controlled study in 100 patients. Part 1: burn wound healing. Burns.

[20] Hundeshagen G, Collins VN, Wurzer P (2018). A prospective, randomized, controlled trial comparing the outpatient treatment of pediatric and adult partial-thickness burns with Suprathel or Mepilex Ag. J Burn Care Res.

[21] Aggarwala S, Harish V, Roberts S (2021). Treatment of partial thickness burns: a prospective, randomized controlled trial comparing four routinely used burns dressings in an ambulatory care setting. J Burn Care Res.

[22] Karlsson M, Steinvall I, Elmasry M (2023). Suprathel® or Mepilex® Ag for treatment of partial thickness burns in children: a case control study. Burns.

[23] Silverstein P, Heimbach D, Meites H (2011). An open, parallel, randomized, comparative, multicenter study to evaluate the cost-effectiveness, performance, tolerance, and safety of a silver-containing soft silicone foam dressing (intervention) vs silver sulfadiazine cream. J Burn Care Res.

[24] Atiyeh BS, Costagliola M, Hayek SN, Dibo SA (2007). Effect of silver on burn wound infection control and healing: review of the literature. Burns.

[25] Storm-Versloot MN, Vos CG, Ubbink DT, Vermeulen H (2010). Topical silver for preventing wound infection. Cochrane Database Syst Rev.

[26] Kokawa A, Yamaguchi H, Tachimori Y, Kato H, Watanabe H, Nakanishi Y (2001). Other primary cancers occurring after treatment of superficial oesophageal cancer. Br J Surg.

[27] Hyland EJ, D'Cruz R, Menon S (2018). Biobrane™ versus acticoat™ for the treatment of mid-dermal pediatric burns: a prospective randomized controlled pilot study. Int J Burns Trauma.

[28] Lal S, Barrow RE, Wolf SE, Chinkes DL, Hart DW, Heggers JP, Herndon DN (2000). Biobrane improves wound healing in burned children without increased risk of infection. Shock.

[29] Barret JP, Dziewulski P, Ramzy PI, Wolf SE, Desai MH, Herndon DN (2000). Biobrane versus 1% silver sulfadiazine in second-degree pediatric burns. Plast Reconstr Surg.

[30] Cassidy C, St Peter SD, Lacey S, Beery M, Ward-Smith P, Sharp RJ, Ostlie DJ (2005). Biobrane versus duoderm for the treatment of intermediate thickness burns in children: a prospective, randomized trial. Burns.

[31] Lesher AP, Curry RH, Evans J (2011). Effectiveness of Biobrane for treatment of partial-thickness burns in children. J Pediatr Surg.

[32] Mandal A (2007). Paediatric partial-thickness scald burns--is Biobrane the best treatment available?. Int Wound J.

[33] Gerding RL, Emerman CL, Effron D, Lukens T, Imbembo AL, Fratianne RB (1990). Outpatient management of partial-thickness burns: Biobrane versus 1% silver sulfadiazine. Ann Emerg Med.

[34] Fan C, Pek CH, Por YC, Lim GJ (2018). Biobrane dressing for paediatric burns in Singapore: a retrospective review. Singapore Med J.

[35] Sheridan RL, Hurley J, Smith MA (1995). The acutely burned hand: management and outcome based on a ten-year experience with 1047 acute hand burns. J Trauma.

[36] Smith MA, Munster AM, Spence RJ (1998). Burns of the hand and upper limb--a review. Burns.

[37] Moore ML, Dewey WS, Richard RL (2009). Rehabilitation of the burned hand. Hand Clin.

[38] Rrecaj S, Hysenaj H, Martinaj M, Murtezani A, Ibrahimi-Kacuri D, Haxhiu B, Buja Z (2015). Outcome of physical therapy and splinting in hand burns injury: our last four years’ experience. Mater Sociomed.

[39] Tan E, Lee HJ, Chong SJ (2017). Use of Biobrane glove finger sleeves on nonintended burn wounds of the hand—a cost-saving method. J Hand Microsurg.

[40] Kok K, Georgeu GA, Wilson VY (2006). The Acticoat glove-an effective dressing for the completely burnt hand: how we do it. Burns.

[41] Ratliff R, King A, Lineaweaver W (2022). Glove dressing for initial management of hand burns. Ann Plast Surg.

[42] Richard R, Baryza MJ, Carr JA (2009). Burn rehabilitation and research: proceedings of a consensus summit. J Burn Care Res.

[43] Esselman PC (2007). Burn rehabilitation: an overview. Arch Phys Med Rehabil.

[44] Cubison TC, Pape SA, Parkhouse N (2006). Evidence for the link between healing time and the development of hypertrophic scars (HTS) in paediatric burns due to scald injury. Burns.

[45] Deitch EA, Wheelahan TM, Rose MP, Clothier J, Cotter J (1983). Hypertrophic burn scars: analysis of variables. J Trauma.

[46] Gangemi EN, Gregori D, Berchialla P (2008). Epidemiology and risk factors for pathologic scarring after burn wounds. Arch Facial Plast Surg.

[47] Chipp E, Charles L, Thomas C, Whiting K, Moiemen N, Wilson Y (2017). A prospective study of time to healing and hypertrophic scarring in paediatric burns: every day counts. Burns Trauma.

[48] Sullivan T, Smith J, Kermode J, McIver E, Courtemanche DJ (1990). Rating the burn scar. J Burn Care Rehabil.

[49] Draaijers LJ, Tempelman FR, Botman YA, Tuinebreijer WE, Middelkoop E, Kreis RW, van Zuijlen PP (2004). The patient and observer scar assessment scale: a reliable and feasible tool for scar evaluation. Plast Reconstr Surg.

[50] Tyack Z, Simons M, Spinks A, Wasiak J (2012). A systematic review of the quality of burn scar rating scales for clinical and research use. Burns.

[51] Richardson P, Mustard L (2009). The management of pain in the burns unit. Burns.

[52] Summer GJ, Puntillo KA, Miaskowski C, Green PG, Levine JD (2007). Burn injury pain: the continuing challenge. J Pain.

[53] Romanowski KS, Carson J, Pape K (2020). American Burn Association guidelines on the management of acute pain in the adult burn patient: a review of the literature, a compilation of expert opinion and next steps. J Burn Care Res.

[54] Church D, Elsayed S, Reid O, Winston B, Lindsay R (2006). Burn wound infections. Clin Microbiol Rev.

[55] Pruitt BA Jr, McManus AT, Kim SH, Goodwin CW (1998). Burn wound infections: current status. World J Surg.

